# Rapid Respiratory Microbiological Point-of-Care Testing and Antibiotic Use in Primary Care: A Randomized Clinical Trial

**DOI:** 10.1001/jamainternmed.2026.1426

**Published:** 2026-05-18

**Authors:** Alastair D. Hay, Samantha Abbs, Matthew Ridd, Stephen Granier, J. Athene Lane, Peter Muir, Jodi Taylor, Grace Young, Kathy Eastwood, Hayley Dash, Lynne Bradshaw, Rebecca Clarke, Mandy Lui, Emma Bridgeman, Rachel C. M. Brierley, Emily Brown, Hannah V. Thornton, Paul M. Mitchell, Liang Zhu, Lucy Yardley, Chris Metcalfe

**Affiliations:** 1 Centre for Academic Primary Care, Bristol Medical School, Population Health Sciences, University of Bristol, Bristol, United Kingdom; 2 Bristol Trials Centre, Bristol Medical School, University of Bristol, Bristol, United Kingdom; 3 Centre for Applied Excellence in Skin and Allergy Research, University of Bristol, Bristol, United Kingdom; 4 Whiteladies Medical Group, Bristol, United Kingdom; 5 UK Health Security Agency South West Regional Laboratory, Southmead Hospital, Bristol, United Kingdom; 6 Bristol, United Kingdom; 7 School of Psychological Science, University of Bristol, Bristol, United Kingdom; 8 Gloucestershire Hospitals NHS Foundation Trust, Gloucester, United Kingdom; 9 Health Economics and Health Policy, Population Health Sciences, Bristol Medical School, University of Bristol, Bristol, United Kingdom; 10 School of Psychology, University of Southampton, Southampton, United Kingdom

## Abstract

**IMPORTANCE:**

Most antibiotic prescribing takes place in primary care, driving antimicrobial resistance, a top-10 threat to global public health. There is considerable international interest in whether rapid multiplex microbiological point-of-care testing (RM-POCT) can safely reduce antibiotic prescribing in primary care.

**OBJECTIVE:**

To investigate whether the use of a RM-POCT can safely reduce same-day antibiotic prescribing for children and adults presenting to primary care with respiratory infections.

**DESIGN, SETTING, AND PARTICIPANTS:**

This parallel-group randomized clinical trial was conducted at 16 general practices in Southwest England between December 2022 and April 2024. Patients were eligible if they were aged 12 months or older, presented with any clinician-diagnosed acute (≤21 days) respiratory tract infection, and the patient or clinician believed antibiotic treatment was, or might be, necessary. Participants were randomized (1:1) to RM-POCT or usual care. The research team, including those conducting statistical analyses, were unaware of group allocation. Data were analyzed from November 21, 2024, to March 13, 2025.

**INTERVENTION:**

Patients in the intervention group were tested with RM-POCT to indicate the presence or absence of 19 respiratory viral pathogens and 4 atypical bacteria in approximately 45 minutes.

**MAIN OUTCOMES AND MEASURES:**

The primary outcome was same-day antibiotic prescribing. The safety outcome was patient-reported symptom severity on days 2 to 4.

**RESULTS:**

Among 552 included patients (mean [SD] age, 40.0 [21.2] years; 349 [63%] female), 276 were randomized to the intervention group and 276 to usual care. Primary outcome data were available for all participants, and safety outcome data were available in 216 intervention participants (78%) and 203 usual care participants (74%). Same-day antibiotics were prescribed to 124 participants (45%) in each group (odds ratio [OR], 1.00 [95% CI, 0.71 to 1.41]; *P* > .99). Prespecified subgroup analyses showed evidence of differentially reduced antibiotic prescribing in participants from whom a virus was detected (OR, 0.35 [95% CI, 0.20 to 0.63]; *P* for interaction < .001) and those with chronic lung disease (OR, 0.55 [95% CI, 0.28 to 1.09]; *P* for interaction = .046) but not children younger than 16 years (OR, 1.75 [95% CI, 0.64 to 4.74]; *P* for interaction = .24), nor where patients and clinicians disagreed on antibiotic necessity (OR, 1.12 [95% CI, 0.63 to 1.98]; *P* for interaction = .53). There was no difference in symptom severity on days 2 to 4 between groups (difference in means, 0.09 [95% CI, −0.10 to 0.27]; *P* = .36).

**CONCLUSIONS AND RELEVANCE:**

In this randomized clinical trial among patients with respiratory tract infections being considered for antibiotic treatment in primary care, use of an RM-POCT did not reduce same-day antibiotic prescribing or worsen patient outcomes.

**Trial Registration:**

isrctn.org Identifier: ISRCTN16039192.

## Key Points

**Question:**

Can a rapid multiplex microbiological point-of-care test (RM-POCT) safely reduce antibiotic prescribing for respiratory infections in primary care?

**Findings:**

In this randomized clinical trial that included 552 patients assigned to RM-POCT use or usual care, there was no difference in the proportion prescribed same-day antibiotics. Subgroup analyses showed differentially reduced antibiotic prescribing in patients with chronic lung disease and from whom a virus was detected; there was no difference in symptom severity for days 2 to 4.

**Meaning:**

This randomized clinical trial found that use of an RM-POCT did not reduce same-day antibiotic prescribing or worsen symptom severity on days 2 to 4 in patients with respiratory infections in primary care.

## Teaser

This randomized clinical trial investigates whether the use of a rapid multiplex microbiological point-of-care test can safely reduce same-day antibiotic prescribing for children and adults presenting to primary care with respiratory infections.

## Introduction

Point-of-care tests (POCTs) are widely viewed as key to reducing unnecessary antibiotic prescribing,^1,2^ with some policymakers calling for diagnostic testing to precede every prescription.^3,4,5^ There are currently 2 types of POCT available for this use in primary care.^6^ First, host-response tests measure nonspecific acute-phase inflammatory response proteins (such as C-reactive protein and procalcitonin). They have been available for decades, and there is strong evidence that the use of C-reactive protein POCT can safely reduce antibiotic prescribing for people with acute lower respiratory tract infections (RTIs) in primary care.^7,8^

The second type of POCT are rapid microbiological POCTs (RM-POCT), designed to be infection specific. Developed more recently than host-response POCTs, these use polymerase chain reaction (PCR) or antibodies added to lateral flow test gels to detect the presence of microbes or microbe-specific antigens. PCR-based products can test for multiple microbes from a single sample but are more expensive than lateral flow tests, which usually test for no more than 3 microbes. Furthermore, evidence for RM-POCT use in primary care is sparse, particularly for PCR-based testing, with data limited to small observational studies assessing clinical acceptability, diagnostic performance, and turnaround times.^9,10,11^ Despite this lack of evidence, use of RM-POCT has started to appear in clinical pathways, such as group A *Streptococcus* testing for patients with sore throat.^12^ Indeed, the key weakness of RM-POCTs is the assumption that microbial detection equals microbial etiology. While this may be true for uncontaminated samples obtained from sterile sites, it is problematic for the nonsterile sites, such as nose and throat, where microbes like group A *Streptococcus* can be commensal and pathogenic. There is also concern that widespread availability of RM-POCTs (and host-response POCT) could increase demand for scarce primary care appointments.^13^

Therefore, there is a need for research to investigate the role of RM-POCTs.^14^ We conducted what we believe to be the first randomized clinical trial into the efficacy of a multiplex RM-POCT to reduce same-day antibiotic prescribing. Our key secondary clinical objective was to investigate whether use of an RM-POCT changed patients’ symptom severity on days 2 to 4.

## Methods

This randomized clinical trial received ethical approval from the North West–Preston NHS research ethics committee. The trial protocol and statistical analysis plan are provided in Supplement 1 and published elsewhere.^15^ All patients (or caregivers, if the patient was aged <16 years) agreeing to take part completed an electronic or paper consent form. Participants aged 12 to 15 years completed an assent form. This study is reported following the Consolidated Standards of Reporting Trials (CONSORT) reporting guideline.

### Study Design

This was a controlled, parallel-group, randomized clinical trial conducted at 16 general practices in Southwest England (8 each in winters 1 and 2), measuring both antibiotic prescribing and patient-reported outcomes, as recommended for antimicrobial stewardship interventions. In addition to investigating effects on antibiotic prescribing and symptom severity on days 2 to 4, further secondary objectives were to investigate whether use of a RM-POCT changed duration and severity of symptoms, antibiotic prescribing up to day 28, antibiotic consumption, hospital admission for RTIs, patient (or caregiver, if the patient was aged <16 years) intention to consult for future similar illnesses, and health care contacts in the following 6 months.

### Participants

Patients were eligible if they were aged at least 12 months and were presenting to primary care for the first time and within 21 days of onset with a clinician-diagnosed RTI, where the treating clinician or patient believed antibiotic treatment was, or might be, necessary. Patients also had to be willing to have a nasal and throat swab taken, wait for the RM-POCT result before an antibiotic treatment decision, complete a symptom diary for up to 28 days, and agree to data collection from their medical records. Patients were excluded if they were known to have cystic fibrosis, required hospital admission, had previously taken part in the study, or were taking part in a conflicting study.

### Procedures

#### Recruitment

A study champion (ie, a trained receptionist or a member of the clinical team) monitored appointment requests to identify potentially eligible patients between December 2022 and April 2024. Potential participants were offered a participant information sheet and the opportunity to ask questions (eFigure 1 in Supplement 2). The treating clinician assessed the patient (per usual care), confirmed eligibility, and received informed consent (eFigure 1 in Supplement 2). Participants recorded baseline illness characteristics (eAppendix 1 and eAppendix 2 in Supplement 2), and clinicians recorded participant clinical details using an electronic case report form. Race and ethnicity were self-reported and categorized as Asian, Black, White, multiple races, or other race or ethnicity. Race and ethnicity data were collected to assess the representativeness of the final study sample.

#### Randomization and Masking

Following consent, participants completed a baseline questionnaire about their symptoms and provided a Sigma Σ-VIROCULT (MWE) combined nose and throat swab (eFigure 1 in Supplement 2). Swabs were placed in viral transport medium. Then participants were individually randomized 1:1 to intervention (RM-POCT) or usual care (no RM-POCT) (eFigure 1 in Supplement 2). Allocation was concealed by use of an internet-based randomization system, developed and maintained by Sealed Envelope. Randomization was stratified by age (<16 years vs ≥16 years) and chronic lung disease, defined as asthma, chronic obstructive pulmonary disease, emphysema, or bronchiectasis (present vs absent). Participants and clinicians were aware of allocation since trial procedures differed by group. The research team, including those conducting statistical analyses, were unaware of group allocation.

#### Intervention

The intervention involved providing the clinician with results from the portion of viral transport medium tested using the BioFire FilmArray Torch 1 in conjunction with BioFire RP2.1 plus reagent pouches (BioMérieux) (eFigure 1 in Supplement 2). Taking approximately 45 minutes, this indicated the presence or absence of 23 respiratory microbes: 19 viruses (influenza A [no subtype detected, H1, H1-2009, and H3], influenza B, adenovirus, coronaviruses [HKU1, NL63, 229E, OC43, Mers-CoV, and SARS-CoV-2], human metapneumovirus, human rhinovirus or enterovirus, parainfluenza [types 1, 2, 3, and 4], and respiratory syncytial virus); and 4 atypical bacteria (*Bordetella pertussis, B parapertussis, Chlamydia pneumoniae*, and *Mycoplasma pneumoniae*). Sites were provided with instructions on how to process samples, and clinicians were given an information sheet describing the typical presentation of illnesses caused by the microbes tested (eAppendix 3 in Supplement 2). Clinicians were not provided with any guidance or training to guide treatment with the test results or on how to communicate results to patients, since communication skills training has previously been shown to influence antibiotic prescribing,^7^ and we wished to understand the effect of the RM-POCT only. Intervention clinicians were asked to wait for the RM-POCT result before deciding treatment (eFigure 1 in Supplement 2). All remaining viral transport media (from intervention and usual care participants) was sent within 24 hours to the UK Health Security Agency South West Regional Laboratory at Southmead Hospital for identical RM-POCT using the BioFire FilmArray Torch 1 platform. Intervention fidelity was assessed through a manual review by L.B. of the unique test identifiers provided by the RM-POCT, allowing confirmation of the patient identity and timing of testing.

#### End Points

The primary (superiority) end point was same-day antibiotic prescribing (immediate or delayed, where a prescription is provided but the patient is advised to delay pharmacy collection) for RTI (eFigure 1 in Supplement 2), as reported by the treating clinician using the electronic case report form. The key secondary (noninferiority) end point was symptom severity on days 2 to 4 using a validated trial diary^16^ (parts C and D; eAppendix 1 [adults] and eAppendix 2 [children] in Supplement 2). Participants were asked to report the presence and severity of key symptoms daily, from 0 (normal) to 6 (as bad as it could be), until either all symptoms resolved or 28 days.

Other secondary end points were antibiotic and antiviral prescribing within 28 days (primary care medical notes review), consultations for respiratory infections within 6 months (primary care medical notes review), hospital admissions within 28 days (primary care medical notes review), participant-reported antibiotic and antiviral consumption within 28 days (Trial Diary), participant-reported symptom severity and duration and length of time to return to usual activities (Trial Diary), and participant intention to consult for similar future illnesses (questionnaire at 2 months). Adverse event reporting is described in the eMethods in Supplement 2.

### Statistical Analysis

Details of the sample size calculation and prespecified statistical analysis are presented in the trial protocol^15^ and statistical analysis plan in Supplement 1. In brief, assuming an antibiotic prescribing rate of 60% in the usual care group, 244 participants per group would allow a true reduction to 45% in the RM-POCT group to be detected with 90% power at 5% significance. A total randomization target of 514 allowed for 5% attrition. In terms of the key secondary end point, assuming no true difference between groups, 206 participants (80%) completing diaries in each group gave 90% power for a 1-sided 95% CI to exclude increases in the mean symptom score of 20% or more at days 2 to 4.^15^

Analyses of primary and secondary end points were conducted on an intention-to-treat basis, including all participants who provided the necessary measures in their allocated groups. No formal adjustment for multiple tests was made, and the number of statistical tests performed should be considered when interpreting the results of secondary end point measures.

The primary end point measure, prescription of an antibiotic, was compared between allocated groups using a logistic regression model, adjusted for participants’ age (<16 vs ≥16 years) and presence of chronic lung disease. The estimated treatment effect was presented as an odds ratio (OR), 95% CI, and 2-sided *P* value, the latter resulting from a likelihood ratio test.

This analytic approach was adapted to the secondary end point measures. The key secondary end point, mean symptom severity scores on days 2 to 4, was compared between groups using a linear mixed-effects model with participant fitted as a random effect, and days 2 to 4 distinguished by indicator variables. A 1-sided 95% CI is presented, and if it excluded a 20% greater symptom severity score in the RM-POCT group compared with the usual care group, then the RM-POCT group was considered noninferior on this measure.

Prespecified subgroup analyses compared estimated intervention effects on the primary end point between: detection or absence of a virus, presence or absence of chronic lung disease, child and adult participants, and baseline clinician-patient disagreement on antibiotic necessity (defined as the participant believes antibiotics are, or may be, needed and the clinician disagrees). A post hoc subgroup analysis estimated the intervention effect on the key secondary end point between those in whom a virus was and was not detected.

Further details on statistical methods are provided in the eMethods in Supplement 2. We used Stata software version 18 (StataCorp) for all statistical analyses. Data were analyzed from November 21, 2024, to March 13, 2025.

## Results

**Figure 1.**
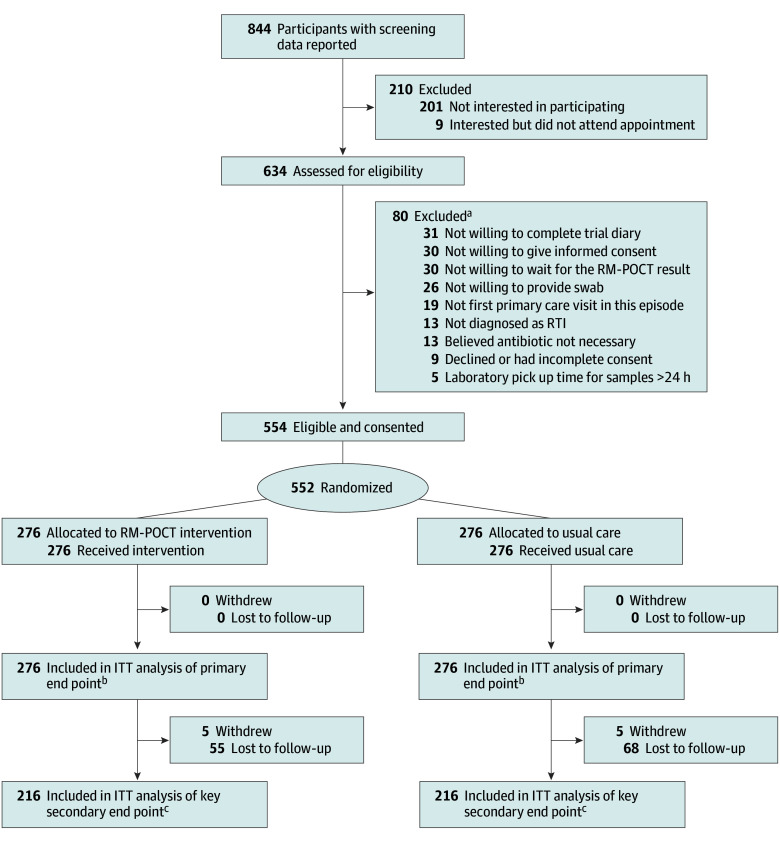
Participant Enrollment Flowchart ITT indicates intention to treat; RTI, respiratory tract infection; RM-POCT, rapid microbiological point-of-care test. ^a^Participants may have been ineligible for more than 1 reason. ^b^Antibiotic prescribing collected on day 1 at appointment 2 (eFigure 1 in Supplement 2). ^c^Symptom severity on days 2 to 4 (eFigure 1 in Supplement 2).

A total of 552 patients (mean [SD] age, 40.0 [21.2] years; 349 [63%] female) from 16 general practices were enrolled and randomized between December 2022 and April 2024, with 276 patients randomized to RM-POCT–informed antibiotic prescribing and 276 patients randomized to usual care (Figure 1). The trial was stopped when symptom severity data were available for 412 participants, as prespecified. Overall, 11 participants (2%) were Asian, 520 participants (94%) were White, and 10 participants (2%) reported multiple races or other race or ethnicity. There were 140 participants (25%) with preexisting chronic lung disease (Table 1). Clinicians diagnosed a wide variety of upper and lower RTIs, typical in routine primary care. Compared to practices nationally, study practices tended to prescribe fewer antibiotics and serve less socioeconomically deprived populations (eTable 1 in Supplement 2).

**Table 1.** Participant Characteristics, Medical History, Clinician Findings and Diagnosis, and Microbiology

Characteristic	Intervention (n = 276)		Usual care (n = 276)	
	No.^a^	No. (%)	No.^a^	No. (%)
Demographic characteristics and medical history				
Sex	276		276	
Female		171 (62)		178 (64)
Male		105 (38)		98 (36)
Age				
Mean (SD), y		41.3 (21.5)		38.7 (20.8)
≥16 y		237 (86)		236 (86)
Race or ethnicity				
Asian		4 (1)		7 (3)
White ethnic group		261 (95)		259 (94)
Multiple or other		5 (2)		5 (2)
Index of Multiple Deprivation quintile				
1 (Most deprived)	272	37 (14)	270	38 (14)
2		30 (11)		31 (11)
3		64 (24)		61 (23)
4		66 (24)		63 (23)
5 (Least deprived)		75 (28)		77 (29)
Chronic lung disease	276	69 (25)	276	71 (26)
Consulted for RTI in past 12 mo		90 (33)		101 (37)
Antibiotics received in the past 12 mo		58 (21)		67 (24)
Positive COVID-19 test result in the past 3 mo		16 (6)		20 (7)
Receiving antibiotics in current illness^b^		1 (<1)		2 (<1)
Examination findings				
Inflamed pharynx or tonsils	185	95 (51)	200	105 (53)
Cervical glands	189	45 (24)	203	47 (23)
Pallor	264	50 (19)	261	47 (18)
Crackles or crepitations	246	52 (21)	247	45 (18)
Wheeze	248	49 (20)	251	36 (14)
Pus on tonsils	186	24 (13)	199	20 (10)
Signs consistent with acute otitis media	97	12 (12)	108	12 (11)
Abnormal pulse^c^	271	43 (16)	269	18 (7)
Abnormal respiratory rate^c^	225	15 (7)	229	16 (7)
Temperature >38 °C	248	16 (6)	255	8 (3)
Bronchial breathing	237	7 (3)	240	3 (1)
Oxygen saturation <94%	265	2 (<1)	262	1 (<1)
Intercostal or subcostal recession	251	1 (<1)	253	0 (0)
Clinician diagnosis and overall assessment				
Common cold	276	32 (17)	276	54 (20)
Acute cough		53 (19)		43 (16)
Acute pharyngitis or tonsilitis		38 (14)		37 (13)
Acute lower respiratory tract infection		39 (14)		21 (8)
Chest infection		28 (10)		23 (8)
Sore throat		27 (10)		22 (8)
Infective exacerbation of chronic lung disease		18 (7)		19 (7)
Influenza		10 (4)		19 (7)
Acute sinusitis		10 (4)		6 (2)
Acute bronchitis		6 (2)		12 (4)
Acute otitis media		4 (2)		12 (4)
Acute laryngitis		6 (2)		6 (2)
COVID-19		3 (1)		0 (0)
Other^d^		2 (<1)		2(<1)
Gut feeling that something is wrong		118 (43)		114 (42)
How unwell do you consider the participant to be, mean (SD) rating^e^		4.3 (2.0)		3.8 (1.9)
Microbiology^f^				
No microbe detected	273^g^	141 (52)	273^g^	138 (51)
Viruses detected, No.				
1		99 (36)		98 (36)
2		12 (4)		17 (6)
3		1 (<1)		1 (<1)
Atypical bacteria detected				
1		18 (7)		11 (4)
Virus and atypical bacteria detected		2 (<1)		8 (3)
Viruses				
Human rhinovirus or enterovirus	273^g^	54 (20)	273^g^	48 (18)
Human metapneumovirus		18 (7)		11 (4)
Parainfluenza virus 3		9 (3)		15 (6)
SARS-CoV-2		6 (2)		18 (7)
Influenza A		12 (4)		11 (4)
Adenovirus		9 (3)		11 (4)
Respiratory syncytial virus		5 (2)		6 (2)
Coronavirus HKU1		2 (<1)		7 (3)
Coronavirus 229E		4 (1)		4 (1)
Coronavirus OC43		2 (<1)		6 (2)
Coronavirus NL63		2 (<1)		5 (2)
Influenza B		2 (<1)		3 (1)
Parainfluenza virus 2		1 (<1)		0 (0)
Parainfluenza virus 4		0		1 (<1)
MERS-CoV		0		0
Parainfluenza virus 1		0		0
Atypical bacteria				
* Mycoplasma pneumoniae*	273^g^	15 (5)	273^g^	10 (4)
* Bordetella pertussis*		1 (<1)		8 (3)
* Bordetella parapertussis*		3 (1)		1 (<1)
* Chlamydia pneumoniae*		1 (<1)		0

Abbreviations: RM-POCT, rapid microbiological point-of-care test; RTI, respiratory tract infection.

^a^ Number of participants providing data for this measure.

^b^ Patients already being treated with antibiotics (for any indication) were included if the treating clinician suspected a new (or ongoing) RTI and the clinician or participant believed further antibiotic treatment was, or may have been, necessary. No patient was already being treated with an antiviral.

^c^ Abnormal pulse and respiratory rate values were defined according to age. For children, thresholds were based on the National Paediatric Early Warning Score charts published by National Health Service England. For adults, a normal pulse rate was defined as 60 to 100 beats per minute and a normal respiratory rate as 12 to 20 breaths per minute, per National Health Service guidance.

^d^ Some free-text responses in the other category have been recategorized to ensure consistency.

^e^ Range, 0 (well) to 10 (very unwell).

^f^ Usual care group results from same RM-POCT in research laboratory.

^g^ Number of results available. Three results were not available due to machine fault and invalid test result in the intervention group. In the usual care group, 2 samples were not received at the laboratory and 1 sample was received at the laboratory but discarded due to labeling issues.

General practitioners and nurses recruited similar numbers of participants (230 participants [42%] and 217 participants [39%], respectively), with paramedics and physician assistants (now part of the UK’s primary care clinician workforce responsible for treating patients with RTIs) recruiting the remainder (66 participants [12%] and 33 participants [6%], respectively) (eTable 2 in Supplement 2). Nearly all participants were recruited at face-to-face consultations, and 552 participants (95%) provided a dual nose-throat swab, taken by site staff on more than 80% of occasions (eTable 2 in Supplement 2).

A valid RM-POCT result was received in 273 intervention participants (99%), and no additional tests were used for usual care participants. When comparing RM-POCT results obtained in general practice (intervention participants) with those obtained from laboratory testing (usual care participants), the proportions from whom no microbe was detected (141 samples [52%] vs 138 samples [51%]), and at least 1 virus or atypical bacterium was detected (238 samples [44%] and 39 samples [7%], respectively) were similar (Table 1). *M pneumoniae* was the atypical bacterium most detected (15 samples [5%]) (Table 1). Primary end point data were available for all participants. Key secondary end point data were available for 215 intervention participants (78%) and 204 usual care participants (74%) (Figure 1).

Same-day antibiotics were prescribed to 124 participants (45%) in each groups (OR, 1.00 [95% CI, 0.71 to 1.41]; *P* > .99) (Table 2). Immediate and delayed prescribing were used in 159 (64.1%) and 89 (35.9%) of 248 participants, respectively, and were also similar across groups. Results were unchanged by prespecified sensitivity analyses. Prespecified subgroup analyses showed differential antibiotic prescribing reductions in participants in whom the RM-POCT detected 1 or more viruses (OR, 0.35 [95% CI, 0.20-0.63]; *P* for interaction < .001) and those with chronic lung disease (OR, 0.55 [95% CI, 0.28-1.09]; *P* for interaction = .046), but not in children (OR, 1.75 [95% CI, 0.64-4.74]; *P* for interaction = .24) or where participants and clinicians disagreed on antibiotic necessity (OR, 1.12 [95% CI, 0.63-1.98]; *P* for interaction = .53) (Table 3).

**Table 2.** Antibiotic Prescribing, Consumption, and Symptom Severity End Points

Measure	No./total No. (%)		OR (95% CI)^a^	*P* value
	Intervention	Usual care		
Day 1 antibiotic prescribing (primary end point)^a^^,^^b^				
All prescribed antibiotics	124/276 (45)	124/276 (45)	1.00 (0.71 to 1.41)	>.99
Immediate	81/124 (65)	78/124 (63)		
Delayed	43/124 (35)	46/124 (37)		
Sensitivity analyses (primary end point)^c^				
Per protocol population^d^	123/273 (45)	123/273 (45)	1.00 (0.71 to 1.41)	>.99
Adjusting for general practices	NA	NA	0.95 (0.66 to 1.37)	.80
Antibiotic prescribing days 2 to 28^e^				
Participants with ≥1 antibiotic^f^	38/270 (14)	32/273 (12)	1.24 (0.75 to 2.06)	.40
Antibiotic consumption days 1 to 28^g^				
Participants consuming any antibiotic	107/236 (45)	99/233 (42)	1.11 (0.78 to 1.62)	.55
Mean symptom severity score (days 2 to 4), mean (SD) [No.]^h^				
Day 2	2.1 (1.1) [231]	2.0 (1.1) [219]	0.09 (−0.10 to 0.27)^i^	.36
Day 3	1.8 (1.1) [220]	1.6 (1.1) [208]		
Day 4	1.5 (1.0) [217]	1.4 (1.1) [205]		
Sensitivity analysis^c^				
Adjusting for general practice	NA	NA	0.09 (−0.10 to 0.27)^i^	.36
Excluding participants at practices with low symptom diary completion rates	NA	NA	0.09 (−0.10 to 0.29)^i^	.35
Imputed missing data^j^				
Day 2	2.2 (1.1) [231]	2.0 (1.1) [219]	0.13 (−0.06 to 0.32)^i^	.17
Day 3	1.8 (1.1) [220]	1.6 (1.1) [208]		
Day 4	1.5 (1.0) [217]	1.4 (1.1) [205]		

Abbreviations: NA, not applicable; OR, odds ratio.

^a^ One intervention participant was prescribed oseltamivir (eFigure 1 in Supplement 2) and reported it was subsequently consumed.

^b^ Clinician reported (eFigure 1 in Supplement 2), ORs and 95% CIs are from a logistic regression model, adjusting for participant age and presence of chronic lung disease.

^c^ No imbalance in baseline variables observed.

^d^ Three participants from the intervention group and 3 participants from the usual care group were excluded due to unavailable or invalid test results.

^e^ Data collected from primary care medical notes review.

^f^ No antiviral treatments were prescribed.

^g^ Participant-reported symptom diary data.

^h^ The upper bound of the 1-sided 95% CI for the difference in symptom severity between groups was 0.24. The mean symptom score over days 2, 3, and 4 in the usual care group (1.7) was used to calculate the 20% noninferiority margin for the between group difference (1.7 × 0.2 = 0.34). Since the upper bound of the CI did not exceed the predefined 20% margin, noninferiority of the point-of-care testing compared with no point-of-care testing was demonstrated.

^i^ Expressed as difference in means (95% CI). All estimates were adjusted for participant age and presence of chronic lung disease, using a mixed linear regression model.

^j^ Missing data were imputed using the individual’s baseline assessment for the intervention group and a score of zero for the control group. If the baseline score was unavailable, the individual’s day 1 score was used; otherwise, a score of zero was assumed.

**Table 3.** Subgroup Analyses

Measure	Same-day antibiotic prescribing, No./total No. (%)		OR (95% CI)^a^	*P* value
	Intervention	Usual care		
**Prespecified subgroup analyses**				
RM-POCT result^a^				
Virus detected	25/112 (22)	53/116 (46)	0.35 (0.20 to 0.63)	<.001^b^
Virus not detected	98/161 (61)	70/157 (45)	1.89 (1.20 to 2.96)	
Chronic lung disease				
Present	33/69 (48)	44/71 (62)	0.55 (0.28 to 1.09)	.046^b^
Absent	91/207 (44)	80/205 (39)	1.23 (0.83 to 1.83)	
Age at baseline, y				
<16	13/39 (33)	9/40 (23)	1.75 (0.64 to 4.74)	.24^b^
≥16	111/237 (47)	115/236 (49)	0.93 (0.65 to 1.33)	
Participant-clinician baseline disagreement on antibiotic necessity^c^				
Yes	35/101 (35)	38/115 (33)	1.12 (0.63 to 1.98)	
No	89/175 (51)	86/161 (53)	0.87 (0.56 to 1.35)	
**Post hoc analysis**				
Symptom severity score by RM-POCT result (usual care group result from laboratory), mean (SD) [No.]^b^				
Virus detected (n = 228)				
Day 2	2.2 (1.1) [94]	2.1 (1.1) [90]	0.11 (−0.18 to 0.39)	.85^d^
Day 3	1.9 (1.1) [90]	1.8 (1.0) [85]		
Day 4	1.6 (1.1) [90]	1.6 (1.1) [84]		
Virus not detected (n = 318)				
Day 2	2.0 (1.0) [135	1.9 (1.1) [127]	0.07 (−0.17 to 0.31)	
Day 3	1.6 (1.0) [129]]	1.5 (1.1) [121]		
Day 4	1.4 (0.9) [126]	1.3 (1.1) [119]		

Abbreviations: OR, odds ratio; RM-POCT, rapid microbiological point-of-care test.

^a^ Three participants from the intervention group and 3 participants from the usual care group were excluded due to unavailable or invalid test results. The usual care group result was from laboratory testing only.

^b^ *P* value for interaction term, testing the null hypothesis of equal odds ratios in the population between the 2 subgroups.

^c^ Participant believes antibiotics are, or maybe, needed to treat the respiratory tract infection and clinician does not believe or is unsure that antibiotics are needed.

^d^ *P* value for the interaction term, testing the null hypothesis that the treatment effect is the same across the 2 subgroups in the population.

**Figure 2.**
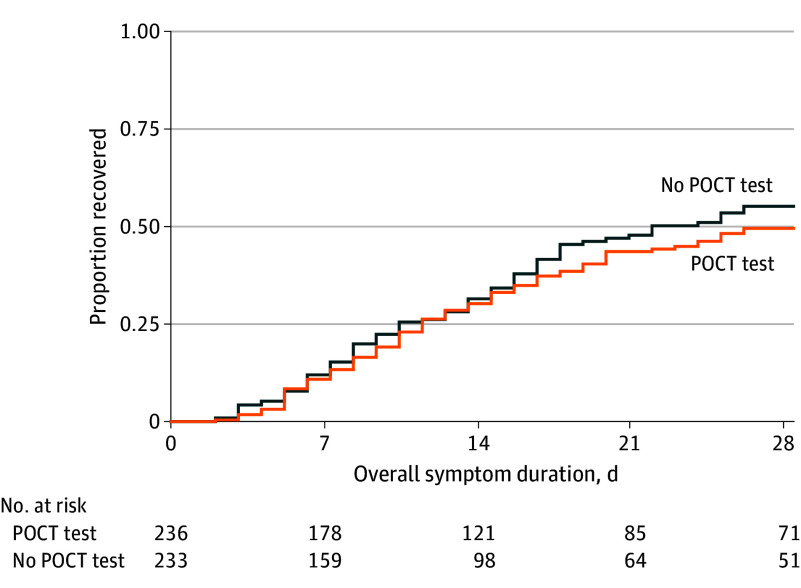
Kaplan-Meier Curve of Overall Symptom Duration by Group POCT indicates point-of-care test.

With regard to the key secondary end point, symptom severity on days 2 to 4, RM-POCT demonstrated noninferiority compared with usual care (difference in means, 0.09 [95% CI −0.10 to 0.27]; *P* = .36) (Table 2) since the upper bound of the CI did not exceed the predefined 20% margin of 0.34 (derived from the mean symptom score over days 2, 3, and 4 in the usual care group of 1.7 × 0.2 = 0.34). A post hoc subgroup analysis showed no evidence of a change in symptom severity from days 2 to 4 in participants with a RM-POCT result positive for a virus (OR, 0.11 [95% CI, −0.18 to 0.39]; *P* for interaction = .85) (Table 3). Figure 2 shows the similarity of the 2 groups with regard to patient-reported overall symptom duration.

Antibiotic prescribing between days 2 and 28 was also similar between the groups (Table 2), and there was no difference in participant reported antibiotic consumption from days one to 28 (Table 2). Figure 2 and eFigures 2 to 4 and eTable 3 in Supplement 2 show the similarity of groups with regard to symptom duration, severity, and rates of second sickening.

There was no evidence that use of the RM-POCT led to an increase in observed health care seeking, either for the index illness or in the subsequent 6 months. The 2-month questionnaire was returned by 66% of the intervention and 58% of the usual care group. These showed no evidence of between-group differences in the proportion intending to consult about similar infections in the future, nor in the proportion wanting a RM-POCT for a similar future illness (eTable 5 in Supplement 2). Two patients in the intervention group and 1 participant in the usual care group were hospitalized for RTI within 28 days of randomization (Table 4 in Supplement 2). Neither was considered related to the study intervention (eTable 7 in Supplement 2).

## Discussion

To our knowledge, this is the first randomized clinical trial of a multiplex RM-POCT in primary care. We found the RM-POCT did not change overall same-day antibiotic prescribing and made no difference to patient-reported symptom outcomes. Interestingly, while antibiotic prescribing halved in the 40% of patients from whom a virus was detected, this reduction was offset by an increase in antibiotic prescribing when no virus or atypical bacteria were detected. We found a high rate of samples with positive results for *M pneumoniae*, coinciding with a UK national outbreak in the 2023 to 2024 winter,^17^ but numbers were too small to understand whether testing and treating improved outcomes in these patients. RM-POCT use did not increase consulting for RTI in the following 6 months, nor did patients express a preference for receiving the same RM-POCT for similar future illnesses.

It is widely believed that most RTIs treated in primary care are viral.^18,19^ However, fewer than half our study patients had test results positive for a virus. This discrepancy may be attributable to enrolling patients for whom antibiotic treatment was considered potentially necessary, use of nasal and throat swabs, and allowing the recruitment of patients with symptoms lasting up to 3 weeks. That said, we found that detecting a virus increased clinician confidence not to prescribe an antibiotic, consistant with the findings of our embedded qualitative research,^20^ and of concern, antibiotic prescribing increased when no microbe was observed. Importantly, this redistribution of antibiotic prescribing did not change patient outcomes. Given the RM-POCT used in this study does not test for typical bacteria, absence of a virus could have been interpreted as presence of a bacterium. This nuanced observation highlights a significant gap in current diagnostic capabilities, emphasizing the need for research to clarify which samples and which POCTs should be used to distinguish between pathogenic and commensal bacteria. However, ultimately, the highest priority is to establish which microbes should be tested for and treated to improve patient outcomes.

Our results are consistent with those investigating similar interventions in secondary care settings. Poehling et al^21^ found a single-plex influenza RM-POCT did not reduce antibiotic prescribing in children with RTIs attending emergency departments, and Mattila et al^22^ found use of another RM-POCT panel (testing for a similar panel of respiratory microbes to the panel used in this study) did not reduce antibiotic prescribing in children with RTIs attending emergency departments.

### Strengths and Limitations

The main study strengths were the use of a randomized controlled design to address a question of importance to policymakers and clinicians internationally, as well as its originality, rigorous conduct, adequate power, intervention adherence, and high follow-up rates. We chose to investigate the efficacy of the RM-POCT only, without including communication skills training (shown to be an effective adjunct to POCT).^7^ We did provide some information regarding the typical presentations for each microbe to help clinician decision-making. Our null result likely reflects the clinical impact if this type of RM-POCT were introduced as-is to routine clinical care. Embedded qualitative research found the RM-POCT was acceptable to clinicians^20^ and patients.^23^ However, additional skills training and guidance on its use may have helped offset the increased use of antibiotics observed when no microbe was detected, and some policymakers would argue that a new diagnostic test should not be provided without training.

In terms of study limitations, the participating general practices had lower antibiotic prescribing rates than the national average and served more affluent and less diverse populations, potentially restricting the generalizability of our findings and possibly explaining the lower-than-expected antibiotic prescribing rate in the usual care group. That said, the wide range of primary care professionals recruiting patients is representative of the primary care workforce. Fewer children were recruited than expected, although recruited patients had a wide range of RTIs for which antibiotics are frequently thought to be necessary. We considered and discounted using nasopharyngeal sampling in favor of nose and throat sampling due to its acceptability during the COVID-19 pandemic. We encountered higher-than-expected attrition in symptom end point measures, possibly resulting in an underestimation of symptom severity and duration, although results were robust to missing data sensitivity analyses and were similar to previous studies.^24^ Although to our knowledge, no similar studies have been conducted, we consider our results are likely to be generalizable to other nations, such as the US, with broadly similar^25,26^ rates of antibiotic prescribing for RTIs.

## Conclusions

This randomized clinical trial examined the effect of RM-POCT testing on antibiotic prescribing for RTIs. Commentators have suggested that primary care clinicians should only prescribe antibiotics after carrying out tests to prove an infection is bacterial^3,5^ and seem to regard RM-POCTs as an antimicrobial stewardship silver bullet.^2,27^ While there is strong trial evidence showing host-response POCTs (such as C-reactive protein) can safely reduce antibiotic prescribing in primary care,^7,8^ our results suggest an RM-POCT that only tests for viruses and atypical bacteria is unlikely to be clinically effective, let alone cost-effective, in primary care. While our null result may be a surprise to some commentators, we suspect the reasons relate to the complexity of the underlying sociopsychobiomedical mechanisms driving antibiotic prescribing, which we investigated in this study and plan to report in future work.

## Notes

Supplement 1. Trial Protocol and Statistical Analysis Plan

Supplement 2. eMethods eFigure 1. Study procedures eAppendix 1. Trial diary (adult) eAppendix 2. Trial diary (child) eAppendix 3. Interpreting BioFire results: information for clinicians eFigure 2. Kaplan-Meier curve of moderately bad, or worse symptom duration by group eFigure 4. Kaplan-Meier curve of number of days to return to usual activity (participants <16 years only) by group eTable 1. Recruitment by GP practice eTable 2. Participant contacts with practice at baseline eTable 3. Symptom duration (secondary end points) eTable 4. Subsequent healthcare contacts and treatment eTable 5. Participant perception of future antibiotic and POCTRM needs eTable 6. Participants formally withdrawing from participation eTable 7. Adverse events eTable 8. Author contributions

Supplement 3. Data Sharing Statement
