# Supplementary material for: Rapid Respiratory Microbiological Point-of-Care Testing and Antibiotic Use in Primary Care: A Randomized Clinical Trial
Source: JAMA Intern Med. 2026 May 18;186(7):817–26. doi: 10.1001/jamainternmed.2026.1426 (PMC13184781; doi:10.1001/jamainternmed.2026.1426)
Supplement: Supplement 2. — eMethods eFigure 1. Study procedures eAppendix 1. Trial diary (adult) eAppendix 2. Trial diary (child) eAppendix 3. Interpreting BioFire results: information for clinicians eFigure 2. Kaplan-Meier curve of moderately bad, or worse symptom duration by group eFigure 4. Kaplan-Meier curve of number of days to return to usual activity (participants <16 years only) by group eTable 1. Recruitment by GP practice eTable 2. Participant contacts with practice at baseline eTable 3. Symptom duration (secondary end points) eTable 4. Subsequent healthcare contacts and treatment eTable 5. Participant perception of future antibiotic and POCTRM needs eTable 6. Participants formally withdrawing from participation eTable 7. Adverse events eTable 8. Author contributions [file jamainternmed-e261426-s002.pdf]

Supplemental Online Content

Hay AD, Abbs S, Ridd M, et al. Rapid respiratory microbiological point-of-care testing and antibiotic use in primary care: a randomized clinical trial. *JAMA Intern Med*. Published online May 18, 2026. doi:10.1001/jamainternmed.2026.1426

eMethods.

eFigure 1. Study procedures

eAppendix 1. Trial diary (adult)

eAppendix 2. Trial diary (child)

eAppendix 3. Interpreting BioFire results: information for clinicians

eFigure 2. Kaplan-Meier curve of moderately bad, or worse symptom duration by group

eFigure 4. Kaplan-Meier curve of number of days to return to usual activity (participants <16 years only) by group

eTable 1. Recruitment by GP practice

eTable 2. Participant contacts with practice at baseline

eTable 3. Symptom duration (secondary end points)

eTable 4. Subsequent healthcare contacts and treatment

eTable 5. Participant perception of future antibiotic and POCTRM needs

eTable 6. Participants formally withdrawing from participation

eTable 7. Adverse events

eTable 8. Author contributions

This supplemental material has been provided by the authors to give readers additional information about their work.

© 2026 Hay AD et al. *JAMA Internal Medicine*.

Methods

Statistical methods

Other symptom end points (overall symptom duration, moderately worse or bad symptom duration and duration of time to return to normal activities) were compared between allocated groups using Cox proportional hazards models and Kaplan-Meier plots. Participants who had not recovered at 28 days were censored.

Sensitivity analyses of the primary end point were repeated with general practices distinguished using indicator variables, and with any baseline variables observed to be imbalanced added to the model, where imbalance was defined as >10 percentage points difference for binary measures or >0.5 standard deviations difference between means for continuous measures.

Sensitivity analyses of mean symptom severity scores on days 2 to 4 were conducted to investigate the potential effect of data being missing not at random. Baseline characteristics of participants included and excluded from the key secondary end point main analysis were presented. The analysis was repeated excluding participants from two practices with diary data completion rates below 60% on days 2 to 4. Additionally, the analysis was repeated with missing data imputed under different assumptions.

Adverse event reporting

Site staff were responsible for recording non-serious adverse events (AEs) assessed as being possibly-, probably- or definitely-related to the intervention or trial procedures and participants were followed up until the event resolved. Sites were also required to record all serious AEs (SAE) and any unexpected SAEs assessed as related to trial procedures were classed as Suspected Unexpected Serious Adverse Reactions (SUSARs).

Role of the funder

The funder had no role in study design, data collection, data analysis, data interpretation, or writing of this report. The intervention (BioFire® FilmArray® Torch 1 platform and BioFire® RP2.1 *plus* reagent pouches) was purchased from BioMérieux™ who had no role in the study design, data collection, data analysis, data interpretation, or writing of this report.

© 2026 Hay AD et al. *JAMA Internal Medicine*.

eFigure 1. Study procedures

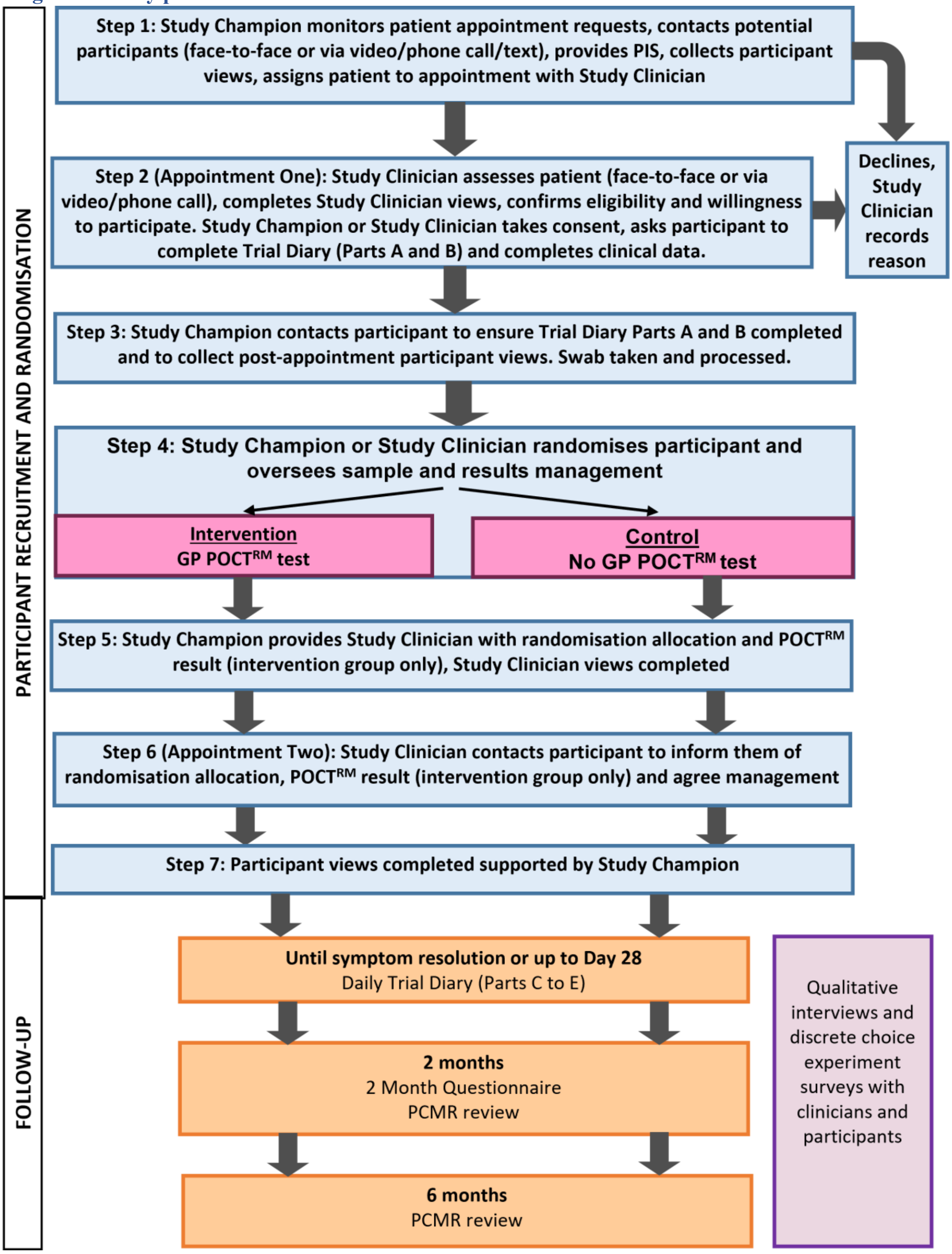

Clinician and participants views refer to a series of questions about perceptions of antibiotic necessity, asked repeatedly to understand if, when and what appeared to change views (to be reported in a future paper).

© 2026 Hay AD et al. *JAMA Internal Medicine*.

eDocument 1. Trial diary (adult)

© 2026 Hay AD et al. *JAMA Internal Medicine*.

Rapid respiratory microbiological point-of-care-testing in primary care (RAPID-TEST trial)

Trial Diary (16 Years and Older)

**Your Participant ID:**<br>(Study Champion to write in and include at the top of each page)	☐☐☐☐☐
**Trial entry date (Day 1):**<br>(Study Champion to write in)	☐☐/☐☐/☐☐☐☐<br>D D M M Y Y Y Y

INSTRUCTIONS FOR COMPLETING THE DIARY

- **Parts A and B** of the diary are for you to complete as soon as the staff at the GP practice ask you to do so. This will be on **Day 1.**
  - Part A is about symptoms related to your current respiratory illness at the start of the trial
  - Part B is for you to rate your general health at the start of the trial

- **Parts C to E** of the diary are for you to complete starting from **the evening of Day 1** up to a maximum of **28 days**.
  - Part C is about symptoms related to your current respiratory illness (to be completed **daily**)
  - Part D is about medicines prescribed for your current respiratory illness (to be completed **daily**)
  - Part E is for you to rate your general health (to be completed **weekly**)

CONTACT US

If you need any help completing the diary or would like to contact the central research team at Bristol Trials Centre, please email us: rapidtest-study@bristol.ac.uk

Thank you for your help with this trial

Participant ID: | | | | | |

PART A: YOUR SYMPTOMS UP TO NOW

1. How unwell do you consider yourself to be today?
   Please tick one box below from 0 (well) to 10 (very unwell).

Well	0	1	2	3	4	5	6	7	8	9	10	Very unwell

2. Duration of current illness (days) ☐☐

3. Has your illness got a lot worse recently? ☐ No ☐ Yes

4. If yes, how many days ago did it start to get worse? ☐☐

5. Complete the table on the next page for symptoms related to your current respiratory illness
   a) Tick **No or Yes** to indicate whether you have had each symptom during this illness
   b) Complete the **score** based on the guidance below to indicate whether you have had each symptom during the past 24 hours

Participant ID: | | | | | |

Guidance: Score each symptom on a scale from 0 to 6 based how you've been feeling over the past 24hrs

0 =	1 =	2 =	3 =	4 =	5 =	6 =
Normal/not affected	Very little problem	Slight problem	Moderately bad	Bad	Very bad	As bad as it could be

**Symptoms**	**Have you had this symptom during your current illness?**		**Enter the score for the past 24 hours (scale 0-6)**
	**No**	**Yes**	
Blocked or runny nose			
Fever (high temperature)			
Cough			
Shortness of breath (breathing faster)			
Phlegm (sputum)			
Wheeze (whistling sound with breathing)			
Eating or drinking less than normal			
Disturbed sleep			
Ear pain			
Change in voice			
Sore throat			
Muscle, facial or head aching or pain			
Sweats or chills			
Unable to do usual activities e.g. work, school, childcare etc			
Other:			
Other:			
Other:			
Other:			

If you have any other symptoms related to your current illness, please write the name of the symptom in the 'Other' box and complete the rest of the row

Participant ID: | | | | | |

<u>PART B: YOUR HEALTH QUESTIONNAIRE (EQ-5D-5L) ON DAY 1</u> <u>(questionnaire not reproduced for copyright reasons)</u>

<u>PART C: SYMPTOMS RELATED TO YOUR CURRENT ILLNESS</u>

- Please complete your symptom scores <u>ONCE A DAY</u> (preferably each evening) in the tables on the next two pages
- Day 1 is the day you entered the RAPID-TEST trial
- **Complete your scores every day until all symptoms are <u>zero for two days in a row</u> or up to Day 28 (whichever comes first)**

Guidance: Score each symptom on a scale from <u>0 to 6</u> based on how you've been feeling over the past 24hrs

0 =	1 =	2 =	3 =	4 =	5 =	6 =
Normal/not affected	Very little problem	Slight problem	Moderately bad	Bad	Very bad	As bad as it could be

Example of how to complete your symptom scores

In the example below, the participant had a runny nose (which was better by Day 12), a fever (which started on Day 3 and was better by Day 12) and a cough (which started on Day 2 and was better by Day 13). They also had loss of smell from Day 1 until Day 11 so entered this in the 'Other' box. Scores were completed until there were two days in a row (Day 13 and Day 14) where all symptoms were zero.

	Symptom Scores			***Symptom Scores***			
	Day 1	***Day 2***	***Day 3***	***Day 11***	***Day 12***	***Day 13***	***Day 14***
Day of the week e.g. M, T, W, Th, F, S, Sun	*M*	*T*	*W*	*Th*	*F*	*S*	*Sun*
Date e.g. 20th Jan	*2nd Feb*	*3rd Feb*	*4th Feb*	*12th Feb*	*13th Feb*	*14th Feb*	*15th Feb*
Blocked or runny nose	*3*	*3*	*2*	*1*	*0*	*0*	*0*
Fever (high temperature)	*0*	*0*	*1*	*1*	*0*	*0*	*0*
Cough	*0*	*2*	*3*	*3*	*2*	*0*	*0*
Other: loss of smell	*1*	*1*	*1*	*1*	*0*	*0*	*0*

If you have any other symptoms related to your current illness, please write the name of the symptom in the 'Other' box and complete the rest of the row

Participant ID: | | | | | |

PART C: Please complete your symptom scores (on a scale of 0-6) based on how you've been feeling over the past 24 hrs for Days 1 to 14

	Symptom Scores													
	**Day 1**	**Day 2**	**Day 3**	**Day 4**	**Day 5**	**Day 6**	**Day 7**	**Day 8**	**Day 9**	**Day 10**	**Day 11**	**Day 12**	**Day 13**	**Day 14**
**Day of the week e.g. M, T, W, Th, F, S, Sun**														
**Date e.g. 20th Jan**														
Blocked or runny nose														
Fever (high temperature)														
Cough														
Shortness of breath (breathing faster)														
Phlegm (sputum)														
Wheeze (whistling sound with breathing)														
Eating or drinking less than normal														
Disturbed sleep														
Ear pain														
Change in voice														
Sore throat														
Muscle, facial or head aching or pain														
Sweats or chills														
Unable to do usual activities e.g. work, school, childcare etc														
Other:														
Other:														
Other:														

Participant ID: | | | | | |

PART C: Please complete your symptom scores (on a scale of 0-6) based on how you've been feeling over the past 24 hrs for Days 15 to 28

	Symptom Scores													
	Day 15	Day 16	Day 17	Day 18	Day 19	Day 20	Day 21	Day 22	Day 23	Day 24	Day 25	Day 26	Day 27	Day 28
**Day of the week e.g. M, T, W, Th, F, S, Sun**														
**Date e.g. 20th Jan**														
Blocked or runny nose														
Fever (high temperature)														
Cough														
Shortness of breath (breathing faster)														
Phlegm (sputum)														
Wheeze														
Eating or drinking less than normal														
Disturbed sleep														
Ear pain														
Change in voice														
Sore throat														
Muscle, facial or head aching or pain														
Sweats or chills														
Unable to do usual activities e.g. work, school, childcare etc														
Other:														
Other:														
Other:														

Participant ID: | | | | | |

PART D: MEDICINES PRESCRIBED FOR YOUR CURRENT ILLNESS

- If you have taken any medicines prescribed for this illness by your doctor or another member of staff at your GP practice e.g. antibiotic or antiviral medicines, please write the name of the medicine and the number of times per day you took each medicine in the tables on the next page.
- **Complete your medicines every day until all symptoms in Part C of this diary are scored as <u>zero for two days in a row</u> or up to Day 28 (whichever comes first)**

Guidance: The names of some antibiotics (also called "antibacterials") that may be used for respiratory infections include:

- Amoxicillin (also called Amoxil)
- Doxycycline (also called Vibramycin)
- Clarithromycin (also called Klaricid)
- Erythromycin (also called Erythrocin)

But there are lots of others.

If you are not sure, please check at https://bnf.nice.org.uk/

If you are unsure if the medicine you are taking is an antibiotic or antiviral please write the name of the medicine in the table anyway

The names of antivirals that may be used for flu and other respiratory infections include:

- Oseltamivir (also called Tamiflu)
- Zanamivir (also called Relenza)
- Amantadine (also called Lysovir)

But there are others. If you are not sure, please check at https://bnf.nice.org.uk/

Example of how to complete your medicines

In the example below, the antibiotic 'amoxicillin' was prescribed 3 times a day for a week. The example shows the participant took amoxicillin 3 times a day on Days 1 to 3 and also on Days 5 to 7 but on Day 4, they only took it twice as one dose was missed.

'Clarithromycin' was taken once on Day 2 and twice on Day 5.

	**Number of Times Medicine Taken Each Day**						
	**Day 1**	**Day 2**	**Day 3**	**Day 4**	**Day 5**	**Day 6**	**Day 7**
Day of the week e.g. M, T, W, Th, F, S, Sun	*M*	*T*	*W*	*Th*	*F*	*S*	*Sun*
Date e.g. 20th Jan	*2nd Feb*	*3rd Feb*	*4th Feb*	*5th Feb*	*6th Feb*	*7th Feb*	*8th Feb*
Amoxicillin	*3*	*3*	*3*	*2*	*3*	*3*	*3*
Clarithromycin	*0*	*1*	*0*	*0*	*2*	*0*	*0*

Participant ID: | | | | | |

PART D: Please complete your medicines for this illness and the number of times you have taken them from Days 1 to 14

	Number of Times Medicine Taken Each Day													
	Day 1	Day 2	Day 3	Day 4	Day 5	Day 6	Day 7	Day 8	Day 9	Day 10	Day 11	Day 12	Day 13	Day 14
Day of the week e.g. M, T, W, Th, F, S, Sun														
Date e.g. 20th Jan														

Enter name of each antibiotic or antiviral medicine you have taken on a separate row

PART D: Please complete your medicines for this illness and the number of times you have taken them from Days 15 to 28

	Number of Times Medicine Taken Each Day													
	Day 15	Day 16	Day 17	Day 18	Day 19	Day 20	Day 21	Day 22	Day 23	Day 24	Day 25	Day 26	Day 27	Day 28
Day of the week e.g. M, T, W, Th, F, S, Sun														
Date e.g. 20th Jan														

PART E: YOUR HEALTH QUESTIONNAIRE (EQ-5D-5L) (questionnaire not reproduced for copyright reasons)

- Complete this health questionnaire at the end of each week. Once all symptoms in Part C of this diary are scored as zero for two days in a row, please complete the health questionnaire at the end of that week and then stop.
  - **Day 7** questionnaire is on pages 12 & 13
  - **Day 14** questionnaire is on pages 14 & 15
  - **Day 21** questionnaire is on pages 16 & 17
  - **Day 28** questionnaire is on pages 18 & 19

RETURNING THE DIARY

Please send the diary back using the FREEPOST envelope provided to:

RAPID-TEST Trial

University of Bristol

Bristol Medical School

Population Health Sciences

1-5 Whiteladies Road

Bristol

BS8 1NU

If you are filling in the diary online, all you need to do is save it as you go along.

Thank you for your help

Acknowledgement and Disclaimer: This project (NIHR131758) is funded by the Efficacy and Mechanism Evaluation (EME) programme, a Medical Research Council (MRC) and National Institute for Health Research (NIHR) partnership. The views expressed in this publication are those of the authors and not necessarily those of the MRC, NIHR or the Department of Health and Social Care.

<insert participant’s email address or postal address>

Bristol Trials Centre
Population Health Sciences
Bristol Medical School
University of Bristol
1-5 Whiteladies Road
Bristol, BS8 1NU

Rapid respiratory microbiological point-of-care-testing in primary care (RAPID-TEST trial)
Trial Diary Reminder

<insert date>

Dear <insert participant’s first name>,

We are writing to follow up on the RAPID-TEST Trial Diary that you were asked to complete for up to 28 days from the day you joined the trial. We understand that you may be very busy just now but would really appreciate it if you could <submit the Trial Diary/return the Trial Diary using the FREEPOST envelope provided> within 2 weeks. Your participation in this trial is important to us.

If you have sent the completed Trial Diary to us in the last few days, please ignore this reminder.

Thank you in advance for your help.

Yours sincerely,

Professor Alastair Hay <insert Clinical Trial
Manager name>
Chief Investigator, RAPID-TEST Trial Clinical
Trial Manager
Tel: <insert
trial phone number>
Email: rapidtest-
study@bristol.ac.uk

eDocument 2. Trial diary (child)

Rapid respiratory microbiological point-of-care-testing in primary care (RAPID-TEST trial)

Trial Diary (Under 16 Years)

Your Participant ID:
(Study Champion to write in and include at the top of each page)

Trial entry date (Day 1):
(Study Champion to write in)

D	D	/	M	M	/	Y	Y	Y	Y

<u>INSTRUCTIONS FOR COMPLETING THE DIARY</u>

- **Parts A and B** of the diary are for you to complete on behalf of your child as soon as the staff at the GP practice ask you to do so. This will be on **<u>Day 1.</u>**
    - Part A is about symptoms related to your child's current respiratory illness at the start of the trial
    - Part B is for you to rate your child's general health at the start of the trial. **This section only needs to be completed for participants aged 4-15 years.**

- **Parts C to E** of the diary are for you to complete on behalf of your child starting from **<u>the evening of Day 1</u>** up to a maximum of **28 days**.
    - Part C is about symptoms related to your child's current respiratory illness (to be completed **daily**)
    - Part D is about medicines prescribed for your child's current respiratory illness (to be completed **daily**)
    - Part E is for you to rate your child's general health (to be completed **weekly**). **This section only needs to be completed for participants aged 4-15 years.**

<u>CONTACT US</u>

If you need any help completing the diary or would like to contact the central research team at Bristol Trials Centre, please email us: rapidtest-study@bristol.ac.uk

Thank you for your help with this trial

PART A: YOUR CHILD'S SYMPTOMS UP TO NOW

6. How unwell do you consider your child to be today?
   Please tick one box below from 0 (well) to 10 (very unwell).

Well	0	1	2	3	4	5	6	7	8	9	10	Very unwell

7. Duration of current illness (days) ☐☐

8. Has your child's illness got a lot worse recently? ☐ No ☐ Yes

9. If yes, how many days ago did it start to get worse? ☐☐

10. Complete the table on the next page for symptoms related to your child's current respiratory illness
    a) Tick **No or Yes** to indicate whether your child has had each symptom during this illness
    b) Complete the **score** based on the guidance below to indicate whether your child has had each symptom during the past 24 hours

Participant ID: | | | | | |

Guidance: Score each symptom on a scale from 0 to 6 based on how your child has been feeling over the past 24hrs

0 =	1 =	2 =	3 =	4 =	5 =	6 =
Normal/not affected	Very little problem	Slight problem	Moderately bad	Bad	Very bad	As bad as it could be

**Symptoms**	**Has your child had this symptom during their current illness?**		**Enter the score for the past 24 hours (scale 0-6)**
	**No**	**Yes**	
Blocked or runny nose			
Fever (high temperature)			
Cough			
Shortness of breath (breathing faster)			
Phlegm (sputum)			
Wheeze (whistling sound with breathing)			
Eating or drinking less than normal			
Disturbed sleep			
Ear pain			
Change in voice/cry			
Child not themselves or more clingy than usual			
Other:			
Other:			
Other:			
Other:			

If your child has any other symptoms related to their current illness, please write the name of the symptom in the 'Other' box and complete the rest of the row

Participant ID: | | | | | |

PART B: YOUR CHILD'S HEALTH QUESTIONNAIRE (Proxy EQ-5D-Y: 1) ON DAY 1

(questionnaire not reproduced for copyright reasons)

PART C: SYMPTOMS RELATED TO YOUR CHILD'S CURRENT ILLNESS

- Please complete your child's symptom scores ONCE A DAY (preferably each evening) in the tables on the next two pages
- Day 1 is the day your child entered the RAPID-TEST trial
- **Complete your child's scores every day until all symptoms are zero for two days in a row or up to Day 28 (whichever comes first)**

Guidance: Score each symptom on a scale from 0 to 6 based on how your child has been feeling over the past 24hrs

0 =	1 =	2 =	3 =	4 =	5 =	6 =
Normal/not affected	Very little problem	Slight problem	Moderately bad	Bad	Very bad	As bad as it could be

Example of how to complete your child's symptom scores

In the example below, the participant had a runny nose (which was better by Day 12), a fever (which started on Day 3 and was better by Day 12) and a cough (which started on Day 2 and was better by Day 13). They also had loss of smell from Day 1 until Day 11 so entered this in the 'Other' box. Scores were completed until there were two days in a row (Day 13 and Day 14) where all symptoms were zero.

	Symptom Scores			***Symptom Scores***			
	Day 1	***Day 2***	***Day 3***	***Day 11***	***Day 12***	***Day 13***	***Day 14***
Day of the week e.g. M, T, W, Th, F, S, Sun	*M*	*T*	*W*	*Th*	*F*	*S*	*Sun*
Date e.g. 20th Jan	*2nd Feb*	*3rd Feb*	*4th Feb*	*12th Feb*	*13th Feb*	*14th Feb*	*15th Feb*
Blocked or runny nose	*3*	*3*	*2*	*1*	*0*	*0*	*0*
Fever (high temperature)	*0*	*0*	*1*	*1*	*0*	*0*	*0*
Cough	*0*	*2*	*3*	*3*	*2*	*0*	*0*
Other: loss of smell	*1*	*1*	*1*	*1*	*0*	*0*	*0*

If your child had any other symptoms related to their current illness, please write the name of the symptom in the 'Other' box and complete the rest of the row

PART C: Please complete your child's symptom scores (on a scale of 0-6) based on how they've been feeling over the past 24 hrs for Days 1 to 14

	Symptom Scores													
	Day 1	Day 2	Day 3	Day 4	Day 5	Day 6	Day 7	Day 8	Day 9	Day 10	Day 11	Day 12	Day 13	Day 14
**Day of the week e.g. M, T, W, Th, F, S, Sun**														
**Date e.g. 20th Jan**														
Blocked or runny nose														
Fever (high temperature)														
Cough														
Shortness of breath (breathing faster)														
Phlegm (sputum)														
Wheeze (whistling sound with breathing)														
Eating or drinking less than normal														
Disturbed sleep														
Ear pain														
Change in voice/cry														
Child not themselves or more clingy than usual														
Other:														
Other:														

Participant ID: | | | | | |

PART C: Please complete your child's symptom scores (on a scale of 0-6) based on how they've been feeling over the past 24 hrs for Days 15 to 28

	Symptom Scores													
	Day 15	Day 16	Day 17	Day 18	Day 19	Day 20	Day 21	Day 22	Day 23	Day 24	Day 25	Day 26	Day 27	Day 28
**Day of the week e.g. M, T, W, Th, F, S, Sun**														
**Date e.g. 20th Jan**														
Blocked or runny nose														
Fever (high temperature)														
Cough														
Shortness of breath (breathing faster)														
Phlegm (sputum)														
Wheeze														
Eating or drinking less than normal														
Disturbed sleep														
Ear pain														
Change in voice/cry														
Child not themselves or more clingy than usual														
Other:														
Other:														
Other:														

PART D: MEDICINES PRESCRIBED FOR YOUR CHILD'S CURRENT ILLNESS

- If your child has taken any medicines prescribed for this illness by their doctor or another member of staff at their GP practice e.g. antibiotic or antiviral medicines, please write the name of the medicine and the number of times per day they took each medicine in the tables on the next page.
- **Complete their medicines every day until all symptoms in Part C of this diary are scored as zero for two days in a row or up to Day 28 (whichever comes first)**

Guidance: The names of some antibiotics (also called "antibacterials") that may be used for respiratory infections include:

- Amoxicillin (also called Amoxil)
- Doxycycline (also called Vibramycin)
- Clarithromycin (also called Klaricid)
- Erythromycin (also called Erythrocin)

But there are lots of others.

If you are not sure, please check at https://bnf.nice.org.uk/

If you are unsure if the medicine your child is taking is an antibiotic or antiviral please write the name of the medicine in the table anyway

The names of antivirals that may be used for flu and other respiratory infections include:

- Oseltamivir (also called Tamiflu)
- Zanamivir (also called Relenza)
- Amantadine (also called Lysovir)

But there are others. If you are not sure, please check at https://bnf.nice.org.uk/

Example of how to complete your child's medicines

In the example below, the antibiotic 'amoxicillin' was prescribed 3 times a day for a week. The example shows the participant took amoxicillin 3 times a day on Days 1 to 3 and also on Days 5 to 7 but on Day 4, they only took it twice as one dose was missed.

'Clarithromycin' was taken once on Day 2 and twice on Day 5.

	**Number of Times Medicine Taken Each Day**						
	**Day 1**	**Day 2**	**Day 3**	**Day 4**	**Day 5**	**Day 6**	**Day 7**
Day of the week e.g. M, T, W, Th, F, S, Sun	*M*	*T*	*W*	*Th*	*F*	*S*	*Sun*
Date e.g. 20th Jan	*2nd Feb*	*3rd Feb*	*4th Feb*	*5th Feb*	*6th Feb*	*7th Feb*	*8th Feb*
Amoxicillin	*3*	*3*	*3*	*2*	*3*	*3*	*3*
Clarithromycin	*0*	*1*	*0*	*0*	*2*	*0*	*0*

Participant ID: | | | | | |

PART D: Please complete your child's medicines for this illness and the number of times they have taken them from Days 1 to 14

	Number of Times Medicine Taken Each Day													
	Day 1	Day 2	Day 3	Day 4	Day 5	Day 6	Day 7	Day 8	Day 9	Day 10	Day 11	Day 12	Day 13	Day 14
Day of the week e.g. M, T, W, Th, F, S, Sun														
Date e.g. 20th Jan														

Enter name of each antibiotic or antiviral medicine they have taken on a separate row

PART D: Please complete your child's medicines for this illness and the number of times they have taken them from Days 15 to 28

	Number of Times Medicine Taken Each Day													
	Day 15	Day 16	Day 17	Day 18	Day 19	Day 20	Day 21	Day 22	Day 23	Day 24	Day 25	Day 26	Day 27	Day 28
Day of the week e.g. M, T, W, Th, F, S, Sun														
Date e.g. 20th Jan														

Participant ID: | | | |

PART E: YOUR CHILD'S HEALTH QUESTIONNAIRE (Proxy EQ-5D-Y: 1)

- Complete this health questionnaire for your child at the end of each week. Once all symptoms in Part C of this diary are scored as zero for two days in a row, please complete the health questionnaire at the end of that week and then stop.
  - **Day 7** questionnaire is on pages 12 & 13
  - **Day 14** questionnaire is on pages 14 & 15
  - **Day 21** questionnaire is on pages 16 & 17
  - **Day 28** questionnaire is on pages 18 & 19
- **Please note: this section only needs to be completed if your child is aged 4-15 years.**

RETURNING THE DIARY

Please send the diary back using the FREEPOST envelope provided to:

RAPID-TEST Trial

University of Bristol

Bristol Medical School

Population Health Sciences

1-5 Whiteladies Road

Bristol

BS8 1NU

If you are filling in the diary online, all you need to do is save it as you go along.

Thank you for your help

Acknowledgement and Disclaimer: This project (NIHR131758) is funded by the Efficacy and Mechanism Evaluation (EME) programme, a Medical Research Council (MRC) and National Institute for Health Research (NIHR) partnership. The views expressed in this publication are those of the authors and not necessarily those of the MRC, NIHR or the Department of Health and Social Care.

JOINTLY FUNDED BY UKRI Medical Research Council NIHR National Institute for Health and Care Research

<insert parent/carer email address or postal address>

Bristol Trials Centre
Population Health Sciences
Bristol Medical School
University of Bristol
1-5 Whiteladies Road
Bristol, BS8 1NU

Rapid respiratory microbiological point-of-care-testing in primary care (RAPID-TEST trial)
Trial Diary Reminder

<insert date>

Dear <insert parent/carer’s first name>,

We are writing to follow up on the RAPID-TEST Trial Diary that you were asked to complete for up to 28 days from the day your child joined the trial. We understand that you may be very busy just now but would really appreciate it if you could <submit the Trial Diary/return the Trial Diary using the FREEPOST envelope provided> within 2 weeks. Your participation in this trial is important to us.

If you have sent the completed Trial Diary to us in the last few days, please ignore this reminder.

Thank you in advance for your help.

Yours sincerely,

Professor Alastair Hay <insert Clinical Trial
Manager name>
Chief Investigator, RAPID-TEST Trial Clinical
Trial Manager
Tel: <insert
trial phone number>
Email: rapidtest-
study@bristol.ac.uk

eDocument 3. Interpreting BioFire results: information for clinicians

Interpreting BioFire Results: Clinician Information Sheet

Background and purpose of this information sheet

1. Primary care clinicians usually make antibiotic prescribing decisions in the absence of any contemporaneous microbiological information.
2. The purpose of the RAPID-TEST trial is to investigate if use of the BioFire® Respiratory panel 2.1 *plus*[1] run on the BioFire® FilmArray® Torch system can reduce same-day antibiotic prescribing in patients aged ≥12 months attending primary care with respiratory tract infections.
3. This sheet aims to provide information to help interpret BioFire results during the RAPID-TEST trial.

What does the BioFire system test, <u>and not test</u>, for?

The BioFire® FilmArray® system detects the presence/absence of the following 23 upper respiratory microbes:

1. 19 viruses
   - Adenovirus
   - Coronaviruses (229E, HKU1, NL63, OC43, MERS-CoV, SARS-CoV-2)
   - Human Metapneumovirus
   - Human Rhinovirus/Enterovirus*
     * Not possible to distinguish due to genetic similarity
   - Influenza A (no subtype detected, A H1, A H3, A H1-2009)
   - Influenza B
   - Parainfluenza (types 1, 2, 3, 4)
   - Respiratory Syncytial Virus
2. Four atypical bacteria
   - *Bordetella parapertussis*
   - *Bordetella pertussis*
   - *Chlamydia pneumoniae*
   - *Mycoplasma pneumoniae*

It **does not** test for the typical respiratory bacteria *S. pneumoniae, S. pyogenes, H. influenzae* or *M. catarrhalis* since these can be commensally carried in the upper respiratory tract.

Clinical presentation associated with microbes

1. Many viral respiratory infections have similar symptoms, commonly including fever, runny/blocked nose, cough (dry and/or productive) and sore throat.
2. **All BioFire detected microbes can cause a wide range of illnesses from mild to severe.**
3. The table below summarises the more specific syndrome presentations associated with some of the BioFire tested microbes.

University of BRISTOL — Rapid-test — BRISTOL TRIALS CENTRE

Table summarising specific syndrome presentations associated with some BioFire tested microbes

Microbe	*Typical presentation*	*Comment*
Viruses		
Influenza viruses	Influenza-like-illness	**Notifiable disease**
SARS-CoV-2	COVID-19	**Notifiable disease**
Respiratory Syncytial Virus	Bronchiolitis in young children, acute lower respiratory infection in others	
Parainfluenza Viruses	Croup in young children	
Rhinovirus	Common cold	
Enterovirus	Very broad range of presentations,[2] including fever, respiratory symptoms, rash, gastroenteritis symptoms and rarely meningitis (usually in children under 3 months)*	
Adenovirus	Conjunctivitis, rash, gastroenteritis, hepatitis (rarely)	
MERS-CoV	Causes Middle East Respiratory Syndrome, a severe respiratory illness in travellers from endemic regions	Seek microbiological advice immediately
Seasonal Coronaviruses	Common cold, sore throat	
Human Metapneumovirus	Common cold, sore throat	
Atypical bacteria		
Mycoplasma pneumoniae	Outbreaks approximately every four years, can be self-limiting/ associated with "wheezy bronchitis" and pneumonia	
Chlamydia pneumoniae	Can be self-limiting/ associated with pneumonia	
Bordetella pertussis	Whooping cough, prolonged cough	**Notifiable disease**
Bordetella parapertussis	Whooping cough, prolonged cough	**Notifiable disease**

* children <12 months excluded from RAPID-TEST trial
** https://www.gov.uk/guidance/notifiable-diseases-and-causative-organisms-how-to-report#list-of-notifiable-diseases

Considerations when interpreting BioFire results

The BioFire result should be used as a guide to clinical decision making, with final responsibility for how the patient is managed resting with the Study Clinician

1. Remember, BioFire **does not** test for the main typical respiratory bacteria (*S. pneumoniae, S. pyogenes, H. influenzae* or *M. catarrhalis* since these can be commensally carried in the upper respiratory tract)
2. Where one or more microbes are detected, you may wish to consider if their presence seems consistent, or inconsistent, with the clinical presentation (see Table)
3. Remember that when a virus or atypical bacterium is identified, co-infection with an unreported typical respiratory bacterium is still possible
4. Diagnostic performance is comparable to laboratory testing with 97.4% overall sensitivity and 99.4% specificity when compared to an FDA-cleared multiplexed respiratory pathogen panel.[1]
5. Results are presented as pathogen detected or not detected. In addition, for Influenza A (including its subtypes) and MERS-CoV only, results may present as equivocal. Please see the BioFire® Respiratory Panel 2.1 *plus* (RP2.1*plus*) brochure (page 19) for more information on the definition of equivocal and the steps to follow with these results.
6. It is common for none, one or multiple (particularly in children) microbes to be detected. There is no evidence to suggest co-infection is associated with more severe illness.

7. False negatives can be due to
    a. Timing of swab sample in relation to illness onset e.g. we know from the COVID pandemic that some patients needed to be tested for SARS-CoV2 multiple times before tests became positive
    b. Poor-quality swab, meaning a microbe being missed
8. False positives can be due to
    a. Commensal carriage – some microbes can reside harmlessly in the upper respiratory tract
    b. Prolonged post-infection "shedding" can occur e.g. rhinovirus can persist for 28 days

Frequently asked questions

1. The BioFire machine tests for a few coronavirus subtypes (HKU1, NL63, 229E, OC43, MERS-CoV, SARS-CoV-2). What difference does this make?
    - SARS-CoV-2 is the cause of COVID-19 and is a notifiable disease.
    - MERS-CoV causes Middle East Respiratory Syndrome, a severe respiratory illness in travellers from endemic region. You should seek microbiological advice immediately.
    - The remaining coronaviruses (HKU1, NL63, 229E, OC43) cause common cold and sore throat symptoms.
2. The BioFire machine tests for Influenza B and a few Influenza A subtypes (no subtype detected, A H1, A H3, A H1-2009). What difference does this make?
    - Influenza A and B can be managed similarly and are notifiable diseases.
    - In patients with a history of avian flu contact, an Influenza A (no subtype detected) positive result *might* indicate the patient has **avian flu**. Seek microbiological advice.
3. The BioFire machine tests for a few Parainfluenza types (1, 2, 3 and 4). What difference does this make?
    - Types 1 and 2 are most common pathogens associated with croup[2]
    - Type 3 are associated with bronchiolitis and pneumonia in young children[2]
    - Type 4 are less common and not well characterised[2]

Comments/suggestions?

We welcome your comments and suggestions to improve this information sheet. Please email rapidtest-study@bristol.ac.uk with "Interpreting BioFire results" in the subject heading.

Authored and reviewed by

Alastair Hay (GP and Professor of Primary Care); Peter Muir (Consultant Virologist); Lucy Yardley (Professor of Health Psychology); Matthew Ridd (GP and Professor of Primary Care); Karen Jordan-Coffee (bioMérieux) and Tom Newcombe (bioMérieux).

References

1. BioFire. BioFire® Respiratory Panel 2.1 *plus*: Biomerieux, 2020.
2. Pediatrics AAo. Red Book: 2003 Report of the Committee on Infectious Diseases. 26th ed. Elk Grove Village, IL.

eFigure 2. Kaplan-Meier curve of moderately bad, or worse symptom duration by group

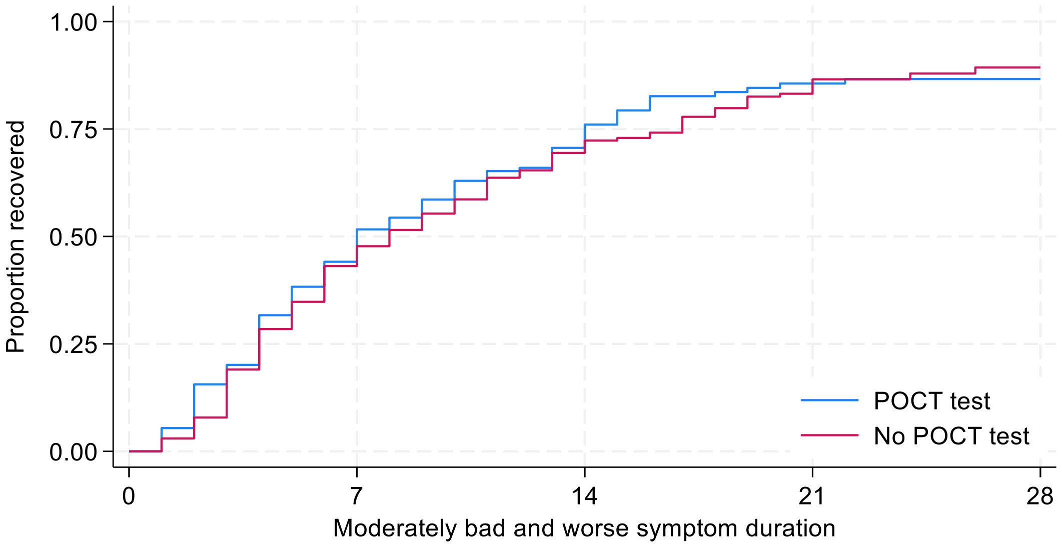

Number at risk					
POCT test	233	89	38	14	11
No POCT test	236	111	53	25	11

eFigure 3. Kaplan-Meier curve of number of days to return to usual activity (participants ≥16 years only) by group

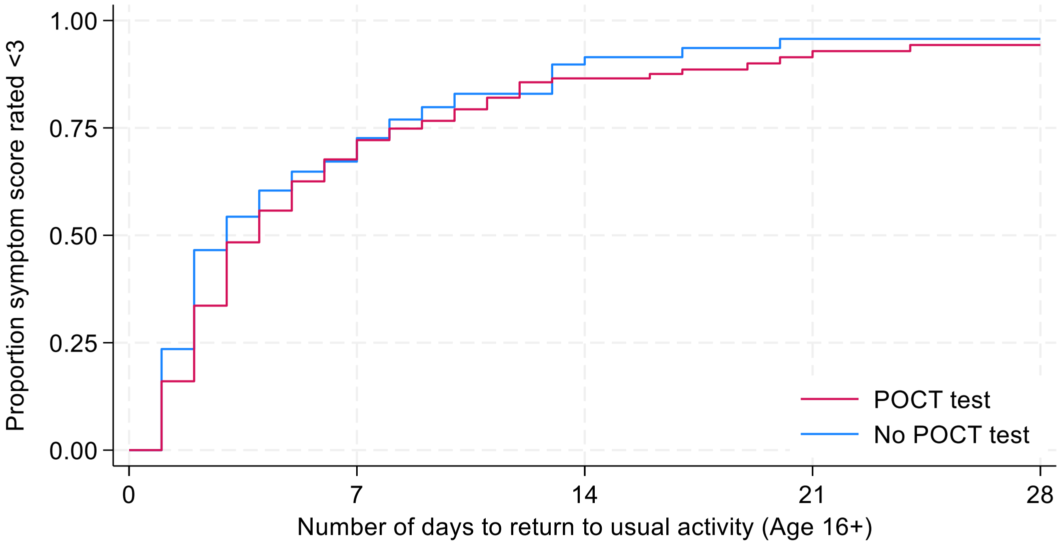

Number at risk					
POCT test	131	36	14	6	3
No POCT test	119	24	6	2	2

eFigure 4. Kaplan-Meier curve of number of days to return to usual activity (participants <16 years only) by group

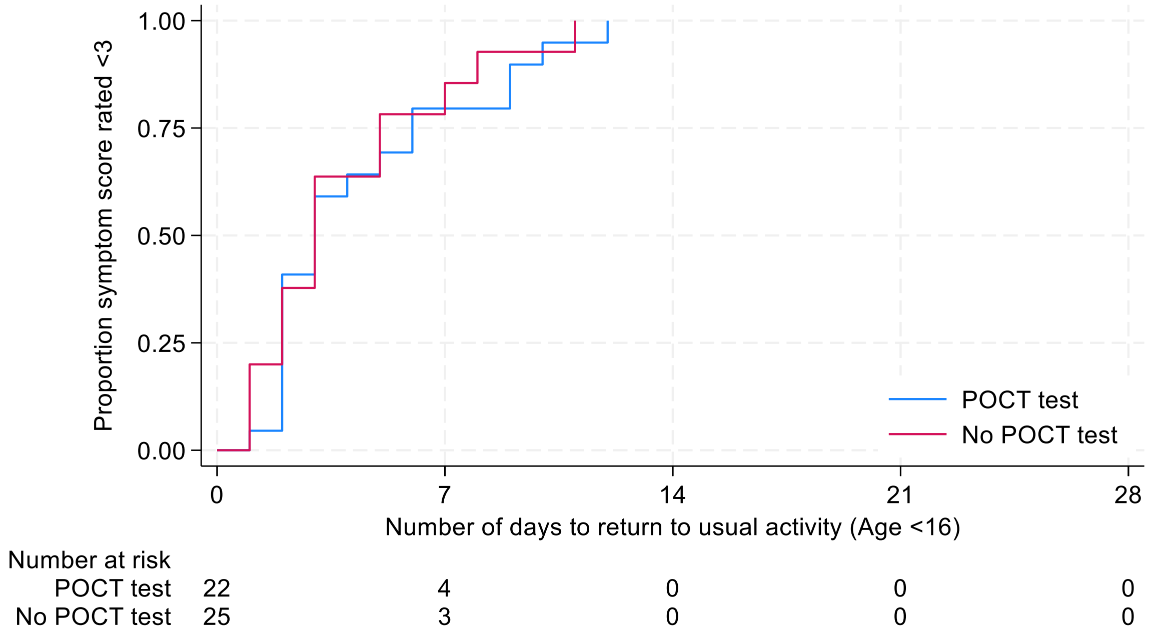

eTable 1. Recruitment by GP practice

GP practices	Intervention (n=276)	Usual care (n=276)	Antibiotic prescribing per 1000 patients per quarter at baseline[a]	UK average in given quarter[a]	Index of Multiple Deprivation (IMD) decile
	Mean (SD) / n (%)	Mean (SD) / n (%)			
Winter 1					
Site A	8 (3%)	17 (6%)	127.6	149.9	7
Site B	19 (7%)	28 (10%)	74.9	107.7	5
Site C	16 (6%)	7 (3%)	130.9	149.9	9
Site D	26 (9%)	27 (10%)	60.0	107.7	9
Site E	28 (10%)	26 (9%)	134.5	149.9	6
Site F	1 (<1%)	1 (<1%)	128.1	149.9	10
Site G	22 (8%)	24 (9%)	168.7	149.9	1
Site H	21 (7%)	11 (4%)	141.4	149.9	9
Winter 2					
Site K	44 (16%)	37 (13%)	101.3	109.1	4
Site L	8 (3%)	5 (2%)	113.2	109.1	7
Site M	13 (5%)	17 (6%)	81.0	109.1	6
Site N	14 (5%)	21 (8%)	107.6	109.1	9
Site P	31 (11%)	31 (11%)	138.2	106.2	7
Site Q	11 (4%)	12 (4%)	116.2	106.2	8
Site R	6 (2%)	2 (1%)	101.0	106.2	5
Site T	8 (3%)	10 (4%)	139.5	129.1	1

[a] Quarter prior to starting recruitment (since practices were recruited sequentially, within and between winters, and therefore started recruitment at different times).

eTable 2. Participant contacts with practice at baseline

	Intervention (n=276)		Usual care (n=276)	
	N[a]	Mean (SD) / n (%)	N[a]	Mean (SD) / n (%)
Clinician profession				
GP	276	115 (42%)	276	115 (42%)
Practice Nurse		90 (33%)		87 (32%)
Paramedic		37 (13%)		29 (11%)
Advance Nurse Practitioner		16 (6%)		24 (9%)
Physician Assistant		15 (5%)		18 (7%)
Pharmacist		2 (<1%)		1 (<1%)
Other[b]		1 (<1%)		2 (<1%)
Consultation type				
Face to face	276	272 (99%)	276	276 (100%)
Phone		4 (1%)		0 (0%)
Videoconference		0 (0%)		0 (0%)
Swab taken				
Throat and nasal	276	262 (95%)	276	260 (94%)
Nasal sample only		8 (3%)		11 (4%)
Throat sample only		6 (2%)		4 (1%)
Not known		0 (0%)		1 (>1%)
Swab taken by				
Study Champion	276	163 (59%)	276	164 (59%)
Study Clinician		62 (22%)		65 (24%)
Participant		47 (17%)		41 (15%)
Parent or carer		4 (1%)		6 (2%)

[a] Number of participants providing data for this measure
[b] Some free-text responses in the 'Other' category have been recategorized to ensure consistency

eTable 3. Symptom duration (secondary end points)

	Number of participants recovered by day 28		Hazard ratio[a] (95% confidence interval)	P value
	Intervention n/N (%)	Usual care n/N (%)		
Overall symptoms[b]	90/236 (38%)	87/233 (37%)	0.89 (0.66, 1.19)	0.431
Moderately bad, or worse, symptoms[c]	171/236 (72%)	143/233 (61%)	0.95 (0.76, 1.19)	0.665
Return to usual activities (adults ≥16 years)[d]	111/200 (56%)	92/197 (47%)	0.89 (0.67, 1.17)	0.396
Return to usual activities (children <16 years)[d]	19/36 (53%)	21/36 (58%)	1.03 (0.63, 1.70)	0.909
	**n/N (%)**	**n/N (%)**	**Odds ratio[a] (95% confidence interval)**	**P value**
Participant experiences worsening symptoms [e]	110/276 (40%)	103/276 (37%)	1.12 (0.79, 1.58)	0.527

[a] All estimates adjusted for participant age and presence of chronic lung disease

[b] The longest number of consecutive days, which one or more symptoms are rated >0, concluding with two days where all symptoms are rated 0

[c] Longest continuous spell (in days) with one or more symptoms rated ≥3, concluding with two days where all symptoms are rated <3

[d] For participants aged ≥16 years, "unable to do usual activities" rated ≥3 on Day 1 should conclude after two consecutive days where the rating is <3. A similar approach applies to participants <16 years old for the question "child not themselves / more clingy than usual"

[e] Over days 2 to 28, the participant sees one or more symptoms increase from score <3 to a score of ≥3 for at least one of the subsequent two days.

eTable 4. Subsequent healthcare contacts and treatment

		Intervention n/N (%) (n=276)	Usual care n/N (%) (n=276)	Odds ratio[a] (95% confidence interval)	P value
Number (%) of participants reconsulting in primary care at least once for a respiratory tract infection up to day 28[b]		65/270 (24%)	57/273 (21%)	1.21 (0.81, 1.81)	0.360
Number (%) of respiratory tract infection consultations per patient between day 29 and 6 months[c]	0	216/269 (80%)	213/273 (78%)	0.90 (0.59, 1.36)	0.615
	1	41/269 (15%)	51/273 (19%)		
	≥2	12/269 (4%)	9/273 (3%)		
Number (%) of participants hospitalized at least once for a respiratory tract infection up to day 28[c]		2/270 (1%)	1/273 (<1%)	..	..

[a] All estimates adjusted for participant age and chronic lung disease status
[b] Data collected from primary care medical records
[C] Participants' primary care medical records were reviewed at 2 months after randomisation

eTable 5. Participant perception of future antibiotic and POCTRM needs[a]

If I (my child) have an infection in future like the one I had when I joined this trial then I …		Intervention[b] n (%)	Usual care[b] n (%)	P value[c]
… will see my doctor to check if antibiotics are needed[d]	Strongly agree	78 (43%)	58 (36%)	0.106
	Agree	67 (37%)	61 (38%)	
	Neither agree nor disagree	20 (11%)	23 (14%)	
	Disagree	6 (3%)	10 (6%)	
	Strongly disagree	9 (5%)	8 (5%)	
… would like to have a point-of-care test to check if antibiotics are needed[e]	Strongly agree	76 (42%)	61 (38%)	0.860
	Agree	70 (39%)	66 (41%)	
	Neither agree nor disagree	17 (9%)	17 (11%)	
	Disagree	5 (3%)	6 (4%)	
	Strongly disagree	13 (7%)	10 (6%)	

[a] Questionnaire administered at two months
[b] Three intervention and three usual care group participants excluded due to unavailable or invalid test results
[c] Ordinal logistic regression model, adjusting for baseline participants' views, age and chronic lung disease status
[d] Adjusted for baseline "I believe an antibiotic is needed to treat my (child's) illness"
[e] Adjusted for baseline "A point of care test would help in making the right decision about whether I (my child) needs antibiotics"

eTable 6. Participants formally withdrawing from participation

Reason for withdrawal	Days after randomisation
Intervention group (n=4)	
Consent invalid and patient changed their mind	0
Too much burden	5
Too much burden	48
Admin/logistical reason	286
Comparison group (n=5)	
Changed their mind	4
Unable to obtain valid informed consent	6
Too much burden	8
Unable to obtain valid informed consent	115
Unable to obtain valid informed consent	149

eTable 7. Adverse events

	Intervention (n=276)	Usual care (n=276)
Number of related[a] adverse events		
0 events	276 (100%)	276 (100%)
Number of Serious Adverse events		
0 events	267 (97%)	275 (100%)
1 event[b]	9 (3%)	1 (<1%)
Number of related [a] serious adverse events		
0 events	276 (100%)	276 (100%)
Number of suspected unexpected serious adverse reactions		
0 events	276 (100%)	276 (100%)

[a] Possibly, probably or definitely related to the intervention or trial procedures.

[b] Among participants who experienced one serious adverse event, six were prescribed antibiotics in the intervention group and none in the usual care group. Nine events were classified as serious due to inpatient hospitalisation or prolongation of existing hospitalisation (8,1), three events were related to RTI (2,1). One event was serious due to inpatient hospitalisation and life-threatening condition (1,0)

Participant ID:

eTable 8. Author contributions (see https://credit.niso.org/)

	AH	SA	MR	SG	AL	PM[u]	JT	PM[i]	LZ	GY	KE	HD	LB	RC	ML	EB[ri]	RB	EB[ro]	HT	LY	CM
Conceptualization	L					S													S	S	S
Data curation		E				S	S	S	S				S			E	S				
Formal analysis	S				S			S	S						S						L
Funding acquisition	L		S	S	S	S	S	S		S	S	S								S	S
Investigation	S	L	S	S	S	S	S	S					S			S	S	S	S		S
Methodology	S	S			S	S		S	S												L
Project administration	S	E											S			E	S				
Software		E					S								S	E	S				S
Resources	S	E					S						S			E	E				
Supervision	E	E	S	S	S	S	S			S	S	S	S	S	S	E	E	S	S	S	S
Validation	S														E						E
Visualisation	S														E						E
Writing – first draft	L	S													S						S
Writing – final review	L	S	S	S	S	S	S	S	S	S	S	S	S	S	S	S	S	S	S	S	S
Final approval	L	S	S	S	S	S	S	S	S	S	S	S	S	S	S	S	S	S	S	S	S
Accountable	L	S	S	S	S	S	S	S	S	S	S	S	S	S	S	S	S	S	S	S	S

Shading relates to ICMJE authorship criteria: all authors must make substantial contributions to at least one activities in each of the blue, yellow, green and orange elements.
E=Equal, L=Lead, S=Supporting.
