# Supplementary material for: Rapid Respiratory Microbiological Point-of-Care Testing and Antibiotic Use in Primary Care: A Randomized Clinical Trial
Source: JAMA Intern Med. 2026 May 18;186(7):817–26. doi: 10.1001/jamainternmed.2026.1426 (PMC13184781; doi:10.1001/jamainternmed.2026.1426)
Supplement: Supplement 3. — Data Sharing Statement [file jamainternmed-e261426-s003.pdf]

Data Sharing Statement

Hay. Rapid Respiratory Microbiological Point-of-Care-Testing and Antibiotic Use in Primary Care. *JAMA Intern Med*. Published May 18, 2026. doi:10.1001/jamainternmed.2026.1426

Data
Additional Information: ISRCTN16039192
Data available: Yes
Data types: Deidentified participant data, Data dictionary
How to access data: https://doi.org/10.5523/bris.b8bdiaoniqt12tpjpsjlt0pxh
When available: With publication

Supporting Documents
Document types: Statistical/analytic code
How to access documents: https://doi.org/10.5523/bris.b8bdiaoniqt12tpjpsjlt0pxh
When available: With publication

Additional Information
Who can access the data: Researchers whose proposed data use has been approved
Types of analyses: Specified purpose
Mechanisms of data availability: After approval of a proposal
